# Immune biomarker correlates from a Phase II study of ipilimumab (IPI) with carboplatin and paclitaxel (CP) in patients with unresectable stage III or IV metastatic melanoma (MM)

**DOI:** 10.1186/2051-1426-3-S2-P259

**Published:** 2015-11-04

**Authors:** Réjean Lapointe, Rahima Jamal, Leon van Kempen, Pamela Thebault, Karl Belanger, Jennifer Friedmann, Jean-Pierre Ayoub, Eftihia Cocolakis, Shirin Kazemi, Jeanne Dionne, Caroline Lambert, Huy Le, Cecile Grange, Jean-Francois Cailhier, Alan Spatz, Wilson Miller

**Affiliations:** 1 Université de Montréal / Centre de Recherche du Centre Hospitalier de l'Université de Montreal (CRCHUM), Montreal, PQ, Canada; 2 Centre Hospitalier de Université de Montréal, Montreal, PQ, Canada; 3 Jewish General Hospital, McGill University, Montreal, PQ, Canada; 4 Université de Montréal,, Montreal, PQ, Canada; 5 Jewish General Hospital, McGill University, Montreal, PQ, Canada; 6 Université de Montréal, Montreal, PQ, Canada

## Background

Pivotal studies with anti-CTLA-4 (Ipilimumab or IPI) demonstrated increased overall survival (OS) both as single agent (10.1 months) and with DTIC (11.2 months). We've previously reported an encouraging safety profile with IPI plus CP and early efficacy results. We now report updated 1-year OS and immune biomarker correlates of patient response.

## Methods

30 patients were randomized in a 1:2 ratio to arm A (C (AUC=6) and P (175mg/m2) every 3 weeks × 5 and IPI (3mg/kg) every 3 weeks × 4 starting at week 4) or arm B (similar dosing to arm A except IPI was given on week after CP). Tumor biopsies were collected at screening and week 8, and immune monitoring bloods were collected throughout.

## Results

Median OS was 16.1 months, with a 1-year OS of 56.5% for all patients with no differences between arms. Overall median follow-up was 23.2 months. Best overall response rate (BORR) and disease control rate (DCR) were 26.7% and 56.7% by irRC. BORR in patients whose tumors were wild type for BRAF and NRAS was 44%, compared to 8% in patients with a mutation in BRAF or NRAS. Clinical responses correlated with the abundance of peri and intratumoral CD3+ inflammatory cells in the pretreatment biopsy, but not with CD4/CD8 ratios or CD20 infiltrate. Circulating levels of some chemokines were elevated in non-responders compared to responders. While IPI influenced B cells and monocyte differentiation, this did not correlate with clinical outcome. Lower levels of PD-1 on CD4 and CD8 T cells were observed in responders compared to non-responders.

